# Supplementary material for: Prevalence, incidence and associated factors of pneumonia among severely malnourished children hospitalized in Mulago National Referral Hospital, Uganda
Source: PLoS One. 2025 Sep 30;20(9):e0331698. doi: 10.1371/journal.pone.0331698 (PMC12483204; doi:10.1371/journal.pone.0331698)
Supplement: Attachment — Submitted filename: Response to Reviewers.pdf [file pone.0331698.s006.pdf]

Response to Reviewers comments (Rebuttal letter)

11th July 2025

To: Frederick Quinn

Academic Editor,

PLOS ONE

Re: Response to comments from the editor and reviewers of the manuscript titled **'Prevalence, incidence and associated factors of pneumonia among severely malnourished children hospitalized in a tertiary hospital in Uganda: Mixed design study'(PONE-D-23-36220).**

We would like to thank the editorial team and the reviewers for reviewing our submission of the above referenced manuscript to PLOS ONE journal for consideration. We have given careful consideration to all the comments /questions from the reviewers. Please see my point-by-point responses to the comments below. The line numbers in our responses indicate where the manuscript has been revised.

Response to reviewers' comments

Reviewer #5:

Comment 1: Title: The title is clear and conveys the essence of the authors' work. Although the title states, "….Prevalence, incidence and associated factors of pneumonia among severely malnourished children hospitalized in a tertiary hospital in Uganda: Mixed study design…", It implies that the authors set out to study Severe malnourishment. However, their work specifically describes severe acute malnutrition (SAM). Are these the same? I suggest they stick to the same nomenclature in the title.

Response: Thank you so much for the comment. We would like to clarify that we studied severe acute malnutrition and the burden or risk to developing pneumonia in the face or environment of severe acute malnutrition. This therefore explains the submitted title of our manuscript.

Comment 2: Background: The background and literature is exhaustive and introduces the main research idea adequately.

Response: Thank you so much for the comment

Comment 3: Methods: You state in the title that the study was conducted at a tertiary hospital in Uganda yet you specify here that The ProbiSAM trial was conducted at Mwanamugimu Nutritional Unit Mulago National Referral and Teaching Hospital between 10th March 2014 to 8th July 2015. You also confirm that it serves referral cases from all over the country. Why don't you refer to this in your title?

Response: Thank you so much for the comment. We have revised and stated specifically that the study was conducted at Mulago National Referral Hospital (see details on first page of manuscript).

Comment 4. Whereas the authors state that the average monthly attendance for inpatients was 120 children aged 1 months to 12 years, 75% of whom are aged 6 to 59 months as the basis for their inclusion of data to be analyzed. This reason is weak and a more detailed explanation and explanation for its potential bias due to the arbitrary cutoff is recommended.

Response: Thank you so much for the observation. In our study we used secondary data so it was difficult to influence the inclusion criteria in the study. However, we acknowledged the possibility of selection bias in our discussion (details are in the clean manuscript line number **593 to 596**).

Comment 5: In discussing procedures at Admission, the authors do not describe what was done in this work relative to the parent study. Please confirm and correctly reference if the procedures described have been reported elsewhere especially by the ProbiSAM trial.

Response: Thank you so much for the comment. We have made revisions to include a reference describing details of how procedures were done in the ProbiSAM trial (see line number **183 to 184** clean copy of the manuscript.).

Comment 6: In the title, a Mixed methods design was advance. I expected both qualitative and quantitative study method, I only see the later. Kindly confirm why there is need to qualify your work as ‘mixed methods design’- preferably with a reference.

Response: Thank you so much for this comment and we acknowledge the misuse of the term ‘mixed study design’. We used quantitative data in our study. We have deleted the phrase mixed method study design (see details on title page of the clean manuscript). Mixed study design may involve collecting both qualitative and quantitative data. We collected or used quantitative data only.

Comment 7: Ethical Approval: It is stated that Human Subject’s Approval; was obtained…...Can you confirm if this is intended to mean Ethical Approval?

Response: Thank you so much for the comment. We acknowledge using the word ‘Human Subject’s Approval ‘yet we intended to mean ethical approval. We have made revisions to indicate clearly that ethical approval was obtained for the parent study (Details are in the main manuscript line number **271 to 273).**

Comment 8: Please confirm and state if the Parent study received Institutional Review Board approval in Uganda

Response: Thank you so much for the comment. The parent study “ProbiSAM trial” obtained ethical approval from the institutional review board of Makerere University School of Medicine Research and Ethics Committee (Reference number: REC REF 2013-132) (see details in the clean manuscript line number **273 to 276).** I have included in the attachments the ethical approval letter to confirm this.

Comment 9: It is stated that, "Written informed consent was obtained from all care givers before enrolment into the study". Was this for the Parent study? If so, clearly state it. Also confirm if the minors provided assent and whether consent for use of secondary data was sought and granted during the parent study. Otherwise, a waiver of consent from the REC should have been obtained.

Response: Thank you so much for this comment. We have made revisions to indicate that a written informed consent or accent from minors was obtained for all participants in the parent study (details are in line number **276 to 278**). Our study was secondary data analysis, and we obtained permission from the principal investigator before analysis. In addition, we obtained approval from REC to use secondary data as part of the current study protocol (Reference number: Mak-SOMREC-2021-123). A letter indicating permission to use data from the ProbiSAM trial by principal investigator and approval of the current study protocol has been attached.

Comment 10: Results: In their results the authors do not discuss the role of probiotics which were administered in the parent study to their outcome. This would be a key variable to consider as part of the analysis plan.

Response: Thank you so much for this comment. We acknowledge probiotics could have an effect on our study outcome of pneumonia. However, the purpose of Probiotics was to assess their effect on diarrhea and not pneumonia. The use of Probiotic in our study was not random and therefore we could not sufficiently assess for its effect on our study outcome.

Comment 11: Line 281, States that, "Most of the children were aged below 24 months" Can you qualify this with a statistic if it's important. This generally applies to the entire results section – review the use of generalized and unsupported narratives in the results section.

Response: Thank you so much for the comment. We have made revisions to include the statistic for children less than 24 months to be 87% (see details in line **283 to 284** clean copy of manuscript). In addition, we have made revisions to include statistics for the generalized and unsupported narratives in the results section.

Comment 12: Were there any microbiological tests done to confirm causative agents for pneumonia in addition to MTB?

Response: Thank you for the comment. No microbiological tests were done to confirm causative agents for pneumonia. However, they analyzed for CMV later on bio banked serum of the ProbiSAM samples.

Comment 13: Whereas you discuss mortality as part of your work (line 479), you do not show any mortality outcomes/data as part of the results. Was this data collected?

Response: Thank you for the comment. We acknowledge that we could have used the word mortality in error, however, in line 479 we intended to discuss the odds of being infected with TB as comorbidity not mortality. We have made revisions to indicate this clearly (see details in clean manuscript line number **482 to 484**). Data on mortality was collected in the main trial however we did not discuss it in this study because it was not one of the outcomes we set out to study.

Comment 14: Discussion: The authors' claim for this to be the first study to report incidence in such a large enough sample size is commendable and a strength for their work. Please emphasize the discussion regarding the time the study was conducted in relation to efforts being made to prevent, protect and treat pneumonia to reduce the morbidity and mortality related with it among children less than 5 years by the year 2025. Are your results enough to justify/support your discussion since the work was done between March 2014 and July 2015?

Response: Thank you so much for the comment. Our findings are still valid and relevant despite that the data was collected between 2014 and 2015. The current prevalence of pneumonia among children with SAM ranges from 26% to 38% which is similar to our study findings of 28%. In our discussion, we explained that adequate nutrition is one of the multifaceted approaches to prevent, protect and treat pneumonia. And yet the burden of severe acute malnutrition in our setting is still high 3.2 % according UDHS 2022. Therefore, burden of pneumonia is still high among children with SAM. We have discussed this details in line **number 411 to 419** of the clean manuscript.

Comment 15: Line 443: …is M. tuberculosis (the causative organism of TB…), Please note that M. tuberculosis should be always written underlined or italicized.

Response: Thank you so much for the observation. We have made revisions and italicized mycobacteria tuberculosis (see details in clean copy of the manuscript line number **485**).

Comment 16: Consider, line 444: "Prone to infections like M. tuberculosis because…of the…"

Response: Thank you so much for the comment. We have made revisions to state as suggested (see details clean copy of the manuscript line number **484 to 486).**

Comment 17: Line 446: I suggest you state, "Could be" and not "Should" since this work is only part of the body of evidence. Consider this for the entire discussion section.

Response: Thank you so much for the comment. We acknowledge that we used the word should be instead of could be in error. However, we have made revisions to replace should be with could be since our findings are part of the wider body of evidence.

Comment 18: Line 479: Your discussion of mortality is not supported by your data and results. Please review.

Response: Thank you so much for the observation. We acknowledge that we could have used the word mortality in error, however, in line 479 we intended to discuss the odds of being infected with TB as comorbidity not mortality so we have made revisions in the clean manuscript 9details are in line number **482 to 484)**
