# Supplementary material for: Prevalence, incidence and associated factors of pneumonia among severely malnourished children hospitalized in Mulago National Referral Hospital, Uganda
Source: PLoS One. 2025 Sep 30;20(9):e0331698. doi: 10.1371/journal.pone.0331698 (PMC12483204; doi:10.1371/journal.pone.0331698)
Supplement: Attachment — Submitted filename: PONE-D-23-36220_Review report.docx [file pone.0331698.s001.docx]

**Manuscript Number PONE-D-23-36220
"Prevalence, incidence and associated factors of pneumonia among severely malnourished children hospitalized in a tertiary hospital in Uganda: Mixed study design"**

**Review report**

**Comments to Authors:**

I have read your manuscript with keen interest. The document is well-written and addresses an important tropical paediatric problem—the burden of pneumonia and associated factors in children admitted with severe acute malnutrition. However, I have a few minor comments:

1. There are several typographical errors throughout the document. For example, line 52, consider saying risk of pneumonia, rather than risk of incidence of pneumonia. Line 68, consider ‘pneumonia was associated with 740,180 childhood deaths’, rather than ‘pneumonia killed 740,180 children’. Please correct these.
2. Line 443, consider that ‘M.tuberculosis rather than tuberculosis is one of the causative organisms..’. Please correct.
3. Within Discussion, consider incident pneumonia, rather than incidence of pneumonia. Please correct.
4. Sample size estimation: the statement on what was the sample computed to detect is incomplete. Please clarify.
5. Limitations: consider that the dataset analysed for this study was generated for a different purpose rather than this specific study. Secondly, consider that the study setting is a tertiary hospital, meaning that the findings may not be generalizable to a different setting.

**Comments to the Editor:**

The manuscript is well-written and addresses an important paediatric health problem. The document has several typographical errors that requires correction before acceptance.
