# Supplementary material for: Prevalence, incidence and associated factors of pneumonia among severely malnourished children hospitalized in Mulago National Referral Hospital, Uganda
Source: PLoS One. 2025 Sep 30;20(9):e0331698. doi: 10.1371/journal.pone.0331698 (PMC12483204; doi:10.1371/journal.pone.0331698)
Supplement: Attachment — Submitted filename: Comments.docx [file pone.0331698.s002.docx]

Thank you for choosing very interesting topics. Though there are lots of publication related to this topic.

The manuscript is well organized and analysis was done very meticulously. Several observations are there for this manuscript,

1. We know that young age, any kind of malnutrition, fever etc are risk factors for developing the pneumonia. So, the findings of your manuscript are not exclusive in this field.
2. Important observation is data collection period. It was 10th March 2014 to 8th July 2015. Old data set which might not reflecting the current situation. By this period COVID has changed the scenario. It has changed the economic condition, immunity, disease pattern etc.
3. in Uganda, PCV was launched in 2014. So, situation might be changed in terms of prevalence and incidence by this period.
4. It would be more catchy if we have the mortality data for community acquired and hospital acquired pneumonia cases.
5. **Result:** How can you use “**Tested positive for malaria**” under **clinical characteristic during hospitalization** in Table 3 and 4? It is part of investigation. Fever is one of the presentations of pneumonia, how could it be a risk factor for pneumonia?

Variables selection for identifying the risk factors was not proper.

6. **Result:** In table 3 we have observed that total participants were 289, edema was present in 200 cases. So, we are getting improper WHZ for edematous cases, as weight is more in edematous cases.
7. **Discussion:** The discussion is too long. For both prevalence and incidence almost, similar observations were described twice. So, redundancy could be avoided with proper use of language.
