# Supplementary material for: Prevalence, incidence and associated factors of pneumonia among severely malnourished children hospitalized in Mulago National Referral Hospital, Uganda
Source: PLoS One. 2025 Sep 30;20(9):e0331698. doi: 10.1371/journal.pone.0331698 (PMC12483204; doi:10.1371/journal.pone.0331698)
Supplement: Attachment — Submitted filename: Response to Reviewers (Rebuttal letter).docx [file pone.0331698.s003.docx]

**Response to Reviewers**

To: George Kuryan
Academic Editor
PLOS ONE

**Re:** Response to comments from the editor and reviewers of the manuscript titled **‘Prevalence, incidence and associated factors of pneumonia among severely malnourished children hospitalized in a tertiary hospital in Uganda: Mixed design study’**(PONE-D-23-36220)

We would like to thank the editorial team and the reviewers for reviewing our submission of the above referenced manuscript to PLOS ONE journal for consideration. We have given careful consideration to all the comments /questions from the reviewers.

Please see my point-by-point responses to the comments below. The line numbers in our responses indicate where the manuscript has been revised.

**Response to reviewers’ comments**

**Reviewer #1:** I have read your manuscript with keen interest. The document is well-written and addresses an important tropical paediatric problem—the burden of pneumonia and associated factors in children admitted with severe acute malnutrition. However, I have a few minor comments:

**Comment 1.** There are several typographical errors throughout the document. For example, line 52, consider saying risk of pneumonia, rather than risk of incidence of pneumonia. Line 68, consider ‘pneumonia was associated with 740,180 childhood deaths’, rather than ‘pneumonia killed 740,180 children’. Please correct these.

**Response:** Thank you so much for the comment. We have corrected the typographical errors in the manuscript. The specific errors pointed out in line 52 and 68 have also been addressed

**Comment 2**. Line 443, consider that ‘M.tuberculosis rather than tuberculosis is one of the causative organisms.

**Response:** Thank you so much for the observation. We have made revisions and considered mycobacteria tuberculosis (For details see line **492** of the clean copy of the manuscript)

**Comment 3**. Within Discussion, consider incident pneumonia, rather than incidence of pneumonia.

**Response:** Thank you so much for the observations. We have made revisions as suggested. We have also made similar corrections throughout the document.

**Comment 4**. Sample size estimation: the statement on what was the sample computed to detect is incomplete. Please clarify.

**Response:** Thank you so much for the observations. We have made revisions to clearly indicate what the sample was computed to detect for both prevalence and incidence (Please see details in the manuscript clean copy line **145 to 148** for prevalence and line **150 to154** for incidence).

**Comment 5**. Limitations: consider that the dataset analyzed for this study was generated for a different purpose rather than this specific study. Secondly, consider that the study setting is a tertiary hospital, meaning that the findings may not be generalizable to a different setting.

**Response:** Thank you so much for the comment. We have made revisions to include the limitations. The main limitations of the study include:

1.The dataset analyzed for this study was generated for a different purpose rather than this specific study.

2.The study setting was a tertiary hospital, therefore, our findings may not be generalizable to a different setting (Please see details are in clean copy of the manuscript copy line number **616 to 620**).

**Reviewer #2:** Thank you for choosing very interesting topics. Though there are lots of publication related to this topic. The manuscript is well organized and analysis was done very meticulously. Several observations are there for this manuscript,

**Comment 1.** We know that young age, any kind of malnutrition, fever etc are risk factors for developing the pneumonia. So, the findings of your manuscript are not exclusive in this field.

**Response:** Thank you so much for the comment. We agree some factors like young age, any kind of malnutrition are known risk factors for developing pneumonia in the general pediatric population which includes both the well-nourished and malnourished.

However, this study increases our understanding of issues around pneumonia among children with severe acute malnutrition (SAM) by highlighting the problem of incident pneumonia. Incident pneumonia negatively affects the outcomes of children hospitalized with SAM if not identified and managed promptly. There is hardly any documentation on incident pneumonia and their associated factors among children hospitalized with severe acute malnutrition.

Regarding the prevalence of pneumonia among children with SAM, apart from evidence on prevalence rates and mortality rates due to pneumonia, there is very limited documentation on characteristics of children who present with this common comorbidity pneumonia which is associated with adverse effects like mortality. Therefore, our findings on prevalence and incidence of pneumonia plus their associated factors are key in this special population of severely malnourished children who are at high risk of death.

**Comment 2.** Important observation is data collection period. It was 10th March 2014 to 8th July 2015. Old data set which might not reflecting the current situation. By this period COVID has changed the scenario. It has changed the economic condition, immunity, disease pattern etc.

**Response:** Thank you so much for the comment. We acknowledge COVID 19 caused economic disruptions which led to increased food insecurity in households, disrupted food supply and breast feeding practices contributing to increased levels of malnutrition. In other words, the problem of malnutrition has not improved but potentially made worse. And yet, malnutrition is still an important risk factor for pneumonia among children under 5 years. As such, this study highlights the importance of pneumonia among children with SAM and the urgent attention required. The burden of severe acute malnutrition is still high (3.2%) in our setting according to the recent UDHS 2022, and this means that we still have many children less than five years at risk of pneumonia. Therefore, the findings from our study may be from an old data set done before COVID 19 but the conclusions are similar to the current pneumonia situation. Therefore, results from the current study have significance and clinical implication in daily practice. We have made revisions in the discussion on how COVID 19 relates with findings in the current study. (please see details in the clean copy of manuscript line number **433 to 442).**

**Comment 3**. in Uganda, PCV was launched in 2014. So, situation might be changed in terms of prevalence and incidence by this period.

**Response:** Thank you so much for the comment. There observations that have reported a decline in pneumonia admissions following introduction of PCV vaccination. However, the rate of progress in reducing pneumonia-related deaths has been slower than for other vaccine-preventable diseases. Currently, pneumonia is reported to be the leading cause of death in children under 5 years accounting for 20% of all deaths in this age group. In Uganda, PCV was introduced during the data collection period of the current study. However, pneumonia studies conducted among severely malnourished children after introduction of PCV vaccine in Uganda have reported a high prevalence of pneumonia. Hospital based studies among malnourished children in Mbarara and Jinja hospitals have reported prevalence of 26.8% and 37.6% respectively. This is in agreement with prevalence of 28% in the current study. In addition, the decline reported in pneumonia admissions may not be the same in malnourished children due to presence of other pneumonia-causative organisms that PCV does not protect against such as klebsiella and viral infections like CMV. Furthermore, the recent statistics from UDHS 2022 shows coverage for complete PCV vaccination (PCV3) is 72% leaving many children under 5 at risk of pneumonia. We have made some revisions in the discussion on how introduction of PCV relates with findings in the current study (please see details in the clean copy of manuscript line number **443 to 457**).

**Comment 4**. It would be more catchy if we have the mortality data for community acquired and hospital acquired pneumonia cases.

**Response:** Thank you so much for the comment. Yes, we acknowledge it would be good to report on mortality of both community and hospital acquired pneumonia. However, we stated that the aim of the study was to focus on prevalence, incidence and their associated factors, and we have recommended to explore deaths among children with community acquired pneumonia versus those with hospital-acquired pneumonia.

**Comment 5**. Result: How can you use “Tested positive for malaria” under clinical characteristic during hospitalization in Table 3 and 4? It is part of investigation. Fever is one of the presentations of pneumonia, how could it be a risk factor for pneumonia?
Variables selection for identifying the risk factors was not proper.

**Response:** Thank you so much for the observation. We have moved the variable of tested positive for malaria to the section of investigations. (please see details in the clean copy of manuscript line number **363 to 364** for table 3 **and 397 to 398** for table 4).

We acknowledge that fever is one of the clinical symptoms of pneumonia and removed fever among the risk factors. After removing fever as a risk factor from the model the outcomes were not affected on running the analysis again. The Prevalence ratios and relative risk including their respective confidence intervals remained similar. (please see details in the results section clean copy of the manuscript line **number 363 to 364** for table 3 and line **397 to 398 for** table 4).

**Comment 6.** Result: In table 3 we have observed that total participants were 289, edema was present in 200 cases. So, we are getting improper WHZ for edematous cases, as weight is more in edematous cases.

**Response:** Thank you so much for the observation. Children with edema are usually heavier that their counterparts without edema. Because they are heavier their expected WHZ may be higher (>-3SD). Therefore, when making a diagnosis for severe acute malnutrition in this category of children we utilize presence of edema not WHZ. From our findings in table 3 the WHZ for those in the category of >-3SD is higher (59%) which is similar to those who had edema present (69%). The difference of 10% may be explained by the different grades of edema. When assessing a child with edema there are different grades (grade 1,2 and 3) basing on the clinical appearance. Therefore, children with grade 1 edema the WHZ may be less than <-3SD because amount of edema is far less compared to those having grade 3 edema. This makes the numbers for edema more than those without edema since we did not stratify in the analysis the different categories of edema we lamped them together.

**Comment 6.** Discussion: The discussion is too long. For both prevalence and incidence almost, similar observations were described twice. So, redundancy could be avoided with proper use of language.

**Response:** Thank you so much for the observations. We have made revisions to summarize the discussion section. For example, the findings around stunting and presumptive TB from both prevalence and incident pneumonia have been discussed once. (please see details under discussion section clean copy of the manuscript line **number 474 to 485 for stunting and 486 to 502 for TB**)

**Comments from editor**

**Comment**. PLOS authors have the option to publish the peer review history of their article ([what does this mean?](https://journals.plos.org/plosone/s/editorial-and-peer-review-process#loc-peer-review-history)). If published, this will include your full peer review and any attached files. If you choose “no”, your identity will remain anonymous but your review may still be made public.

**Response:** No

**Comment:** Have the authors made all data underlying the findings in their manuscript fully available?

The [PLOS Data policy](http://www.plosone.org/static/policies.action#sharing) requires authors to make all data underlying the findings described in their manuscript fully available without restriction, with rare exception (please refer to the Data Availability Statement in the manuscript PDF file). The data should be provided as part of the manuscript or its supporting information, or deposited to a public repository. For example, in addition to summary statistics, the data points behind means, medians and variance measures should be available. If there are restrictions on publicly sharing data—e.g. participant privacy or use of data from a third party—those must be specified.

**Response:** Thank you so much for the comment. The data underlying the findings is available upon reasonable request to the principal investigator of the ProbiSAM trial. Details of how to reach the principal investigator regarding issues of data are indicated below.

“The Ugandan act on Data Protection and Privacy and the European act on General Data Protection Regulation do not allow for personal data to be made available to other researchers without prior written approval from relevant institutions and authorities. The Data Protection Officer of the University of Copenhagen can be contacted about data inquiries at dpo@adm. [ku.dk](http://ku.dk/)”
