# Supplementary material for: Prevalence, incidence and associated factors of pneumonia among severely malnourished children hospitalized in Mulago National Referral Hospital, Uganda
Source: PLoS One. 2025 Sep 30;20(9):e0331698. doi: 10.1371/journal.pone.0331698 (PMC12483204; doi:10.1371/journal.pone.0331698)
Supplement: Attachment — Submitted filename: Comments for author.docx [file pone.0331698.s004.docx]

It is a well written manuscript on an important health problem. Few observations are there for the manuscript.

Introduction:

Authors used old statistics related pneumonia and malnutrition. Recent statistics should be used.

Line 78-79 and line 88-89 are providing almost similar information. Redundancy should be avoided.

Method: It is well organized.

Result:

Table 1:organize the values for variable Septicemia.

Discussion:

Line 430-432: Therefore, CXR should be done for all……….these children. This advice should not be applied directly rather author can use judicious selection of candidates for xray.

Line 433: Please correct the l ine.

Still discussion is long. And also there is redundancy of information.
