# Supplementary material for: Prevalence, incidence and associated factors of pneumonia among severely malnourished children hospitalized in Mulago National Referral Hospital, Uganda
Source: PLoS One. 2025 Sep 30;20(9):e0331698. doi: 10.1371/journal.pone.0331698 (PMC12483204; doi:10.1371/journal.pone.0331698)
Supplement: Attachment — Submitted filename: Response to Reviewers.docx [file pone.0331698.s005.docx]

**Response to Reviewers comments (Rebuttal letter)**

To: Frederick Quinn

Academic Editor,

PLOS ONE

**Re:** Response to comments from the editor and reviewers of the manuscript titled **‘Prevalence, incidence and associated factors of pneumonia among severely malnourished children hospitalized in a tertiary hospital in Uganda: Mixed design study’(PONE-D-23-36220).**

We would like to thank the editorial team and the reviewers for reviewing our submission of the above referenced manuscript to PLOS ONE journal for consideration. We have given careful consideration to all the comments /questions from the reviewers. Please see my point-by-point responses to the comments below. The line numbers in our responses indicate where the manuscript has been revised.

**Response to reviewers’ comments**

**Reviewer #2**

It is a well written manuscript on an important health problem. Few observations are there for the manuscript.

**Comment 1:** Introduction: Authors used old statistics related pneumonia and malnutrition. Recent statistics should be used.

Line 78-79 and line 88-89 are providing almost similar information. Redundancy should be avoided

**Response:** Thank you so much for the comment. We have revised the introduction and used the most appropriate and recent statistic in relation to pneumonia and malnutrition. The specific errors pointed out in Line 78-79 and line 88-89 providing almost similar information have also been addressed. Redundant statements have been deleted (for details see line number 77 to 78 and line 85 to 86 of clean copy of the manuscript).

**Comment 2**

Method: It is well organized.

**Response:** Thank you so much for the comment

**Comment 3**

Result:

Table 1: organize the values for variable Septicemia.

**Response:** Thank you so much for the observation. We have revised and organized the values for the variable of septicemia.

**Comment 4**

Discussion:

Line 430-432: Therefore, CXR should be done for all………. these children. This advice should not be applied directly rather author can use judicious selection of candidates for xray.

**Response:** Thank you so much for the comment. We have revised the specific errors pointed out in Line 430-432 providing a judicious advice on which category of children should have their CXR done to improve diagnosis of pneumonia among malnourished children (for details see line 421 to 423 in the clean copy of the revised manuscript).

**Comment 5**: Line 433: Please correct the l ine.

**Response:** Thank you so much for the comment. For specific error in line 433 correction has been made (for details see line number 424 of clean copy of the manuscript).

**Comment 6:** Still discussion is long. And also there is redundancy of information.

**Response:** Thank you so much for the comment. We have made revisions in the discussions to reduce on the redundancy of information and it is shorter than before

**Reviewer #3:**

**Comment 1:** It is a relevant study. The reviewer comments have been addressed. However, the prevalence of any disease with data which is 10 years old is questionable. That aspect of the study may not be relevant and in fact may be misleading - especially since the pandemic and addition of vaccines in the national schedule. Otherwise, this is a well written study.

I would suggest removing the prevalence from the study title and remove the focus from the prevalence.

**Response:** Thank you so much for the comment. I agree indeed that data used is 10 years old. However, the burden of malnutrition in our setting is still high (3.2%) according to the UDHS 2022 and yet malnutrition is still reported to be a risk factor for pneumonia. Recent studies have reported prevalence of pneumonia in malnourished children in our setting to range between 20 to 38% which is similar to our study findings (28%) (details are indicated in line 407 to 409 of the clean copy of the manuscript).

Additionally, even with the introduction of pneumococcal vaccine, the burden of pneumonia in this special population of malnourished children is still prevalent and is similar to what the situation was 10years ago (details are indicated in line 438 to 440 of the clean copy of the manuscript). The current study has also determined specific factors (characteristics) that relate with pneumonia which is still a big burden among malnourished children. Therefore, we still believe that our findings on prevalence still reflect the current situation of pneumonia among malnourished children in our set up.
